# Renal Cystic Disease Proteins Play Critical Roles in the Organization of the Olfactory Epithelium

**DOI:** 10.1371/journal.pone.0019694

**Published:** 2011-05-13

**Authors:** Jennifer L. Pluznick, Diego J. Rodriguez-Gil, Michael Hull, Kavita Mistry, Vincent Gattone, Colin A. Johnson, Scott Weatherbee, Charles A. Greer, Michael J. Caplan

**Affiliations:** 1 Department of Cellular and Molecular Physiology, Yale School of Medicine New Haven, Connecticut, United States of America; 2 Departments of Neurosurgery and Neurobiology, Yale School of Medicine, New Haven, Connecticut, United States of America; 3 Department of Anatomy & Cell Biology, Indiana University School of Medicine, Indianapolis, Indiana, United States of America; 4 Department of Ophthalmology and Neurosciences, Leeds Institute of Molecular Medicine, University of Leeds, Leeds, United Kingdom; 5 Department of Genetics, Yale University School of Medicine, New Haven, Connecticut, United States of America; Tokyo Medical and Dental University, Japan

## Abstract

It was reported that some proteins known to cause renal cystic disease (NPHP6; BBS1, and BBS4) also localize to the olfactory epithelium (OE), and that mutations in these proteins can cause anosmia in addition to renal cystic disease. We demonstrate here that a number of other proteins associated with renal cystic diseases – polycystin 1 and 2 (PC1, PC2), and Meckel-Gruber syndrome 1 and 3 (MKS1, MKS3) – localize to the murine OE. PC1, PC2, MKS1 and MKS3 are all detected in the OE by RT-PCR. We find that MKS3 localizes specifically to dendritic knobs of olfactory sensory neurons (OSNs), while PC1 localizes to both dendritic knobs and cilia of mature OSNs. In mice carrying mutations in *MKS1*, the expression of the olfactory adenylate cyclase (AC3) is substantially reduced. Moreover, in rats with renal cystic disease caused by a mutation in *MKS3*, the laminar organization of the OE is perturbed and there is a reduced expression of components of the odor transduction cascade (G_olf_, AC3) and α-acetylated tubulin. Furthermore, we show with electron microscopy that cilia in *MKS3* mutant animals do not manifest the proper microtubule architecture. Both *MKS1* and *MKS3* mutant animals show no obvious alterations in odor receptor expression. These data show that multiple renal cystic proteins localize to the OE, where we speculate that they work together to regulate aspects of the development, maintenance or physiological activities of cilia.

## Introduction

In renal cystic diseases, the normal ordered structure of the healthy kidney is progressively replaced by fluid filled cysts that can eventually render the kidney unable to function and necessitate renal replacement therapy. The rate of disease progression, as well as the cyst morphology and multiplicity, varies according to the specific disease and mutations. In recent years, however, it was recognized that virtually all genes known to cause renal cystic disease encode proteins that are associated with either the basal body [1]–[3] or the primary cilium [1], [2], [4]–[6]. Thus, renal cystic diseases constitute a subset of the diseases that have been classified as “ciliopathies [6].”

The primary pathology that characterizes renal cystic diseases is typically a progressive decline in renal function due to the presence and growth of renal cysts. However, due to the important and pervasive roles that primary cilia play in other tissues, there are often extra-renal effects, including cyst formation in the liver [7], [8] and the pancreas [8]. In addition, alterations in the central nervous system, such as hydrocephalus [9], occipital meningoencephalocoele and exencephaly [10], have also been reported. The olfactory epithelium (OE) is a highly specialized epithelial tissue, and olfactory sensory neurons (OSNs) possess multiple highly specialized non-motile chemosensory cilia [11]–[13]. However, in the context of ciliopathies, particularly those associated with renal cystic diseases, the OE has received comparatively little attention.

One recent report that examined a potential role for cystic disease proteins in the olfactory system revealed an altered olfactory bulb orientation attributable to the absence of a cystic disease-related protein [14]. We were particularly intrigued by a report [15] that demonstrated that nephrocystin 6 (NPHP6) localizes to the OE, and that mutations in *NPHP6* can cause anosmia in addition to renal cystic disease. Similarly, Bardet-Biedl Syndrome (BBS) proteins, which are encoded by genes associated with another syndrome which can cause renal cysts, have been implicated in anosmia [16]. Consistent with the reports of anosmia, mutations in the genes encoding either *NPHP6* or *BBS* proteins cause defects in OSN ciliary structure and/or in the expression of ciliary proteins [15], [16]. Therefore, the aim of the present work was to build upon these previous reports to determine whether the expression of proteins associated with renal cystic diseases in the OE is a more general phenomenon. To illustrate whether renal cystic disease proteins may play a role in the anatomical organization of the OE, we primarily focused on the role of a particular cystic disease protein, MKS3. We demonstrate here that several mRNAs/proteins associated with renal cystic disease – polycystin 1 (PC1), polycystin 2 (PC2), Meckel-Gruber syndrome 1 (MKS1) and Meckel-Gruber syndrome 3 (MKS3; meckelin) – are also found in the murine OE. Using animal models, we show that the expression of MKS1 and 3 proteins are necessary for proper OE organization and possibly OSN development.

## Methods

### Animal Models

All experiments were conducted in accordance with the policies and procedures of the Yale IACUC (Protocol # 2008-07267 and 2008-10025), the Indiana University IACUC (MD3119), and the National Institutes of Health principles and guidelines for the Care and Use of Laboratory Animals. All genotyping primer sequences are shown in Table S1. All animal models used have been previously described; details of animal models as well as perfusion fixation protocols can be found in the SI.

*MKS3* rats (wistar-wpk) [9], [17], [18] were housed at the Indiana University School of Medicine with a 12 h light cycle, and given free access to food and water. For immunofluorescence experiments, both affected and intralitter phenotypically normal rats were anesthetized with 100 mg/kg sodium pentobarbital and a thoracotomy performed. An intracardiac perfusion with normal saline was followed by 4% paraformaldehyde (PFA) in 0.1 M phosphate buffer. The heads were immersed in PFA and then transferred to phosphate-buffered saline [PBS: 0.1 M phosphate buffer (PB) and 0.9% NaCl, pH 7.4]. After gross dissection of the head to isolate the nasal cavity, heads were decalcified in saturated EDTA, embedded in OCT blocks and 20 µm cryosections were cut. Care was taken to ensure that sections were taken at a similar depth for each animal. For the purpose of EM, rats were perfused for 2–3 min with PBS-Heparin, then for 10 min with 4% PFA and 2% glutaraldehyde in PBS. The heads were then immersed in the fixative for an additional 4 hrs at 4°C, and then stored in PBS at 4°C. Further preparation of tissue for EM is described below.

*PKD1* and *MOR18-2* mice were housed using a 12 hr light cycle at the Yale University School of Medicine, and given free access to food and water. *MOR18-2* mice, originally generated by Bozza, et al [19], were obtained from The Jackson Laboratory (stock #006722). For the PKD1 mouse model, *PKD1^flox/flox^* mice were crossed with *PKD1^+/−^ OMPcre* [20] mice (*PKD1^flox/flox^* mice were a gift from Stefan Somlo; *OMPcre* mice were a gift from Jane Roskams and Stefan Somlo). Progeny were screened by genotyping, and *PKD1^flox/-^ OMPcre* (OE null), and *PKD1^flox/+^ OMPcre* (OE heterozygote) mice were used for subsequent experiments. *PKD1^flox/-^ OMPcre* were apparently healthy with no obvious phenotype upon inspection of intact animals. For the *MOR18-2* mouse model, heterozygotes were mated to produce wild-type and null littermates, identified by genotyping. Both *PKD1* and *MOR18-2* mice were perfusion-fixed, as described above, in order to prepare tissue for immunohistochemistry.

*MKS1* mice were housed at the Yale University School of Medicine, where they were given free access to food and water and housed using a 12 hr light cycle. E18.5 pups were obtained from timed pregnancies in which a pregnant female was euthanized by CO_2_ inhalation followed by cervical dislocation. Pup heads were immersion fixed in 4% PFA for 4 hrs on ice before being set in blocks for cryosections, and tail samples were saved and used for genotyping.

### Generation of MOR18-2 antibody

A novel MOR18-2 antibody was generated in association with Genscript USA Inc. Briefly, a peptide corresponding to the entire C-terminus of MOR 18-2, with an additional N-terminal cysteine (CKTKQIRTRVLAMFKISCDKDIEAGGNT) was synthesized and conjugated to KLH. Two rabbits were then immunized using standard procedures, and after the final bleed, the antibody was affinity-purified against the peptide itself. Although both rabbits produced an antibody which recognized the intended target, initial experiments showed that the antibody from rabbit “B” gave the strongest signal. Thus, this antibody was used for all subsequent experiments. The antibody was characterized by western blot and immunocytochemistry experiments using HEK 293T cells (American Type Culture Collection, ATCC), as well as by staining the OE of MOR18-2 wild-type and null mice (Figure S5). For these experiments, HEK 293T cell culture, transfection, immunocytochemistry [21] and western blot experiments [22] were all performed as described previously. Immunohistochemistry procedures are described below. The MOR18-2 antibody was used at a dilution of 1∶400 for all immunohistochemistry experiments.

### Isolation of RNA from OE and RT-PCR

Wild-type mice (P21) were euthanized by CO_2_ inhalation, and the OE was rapidly dissected and RNA isolated. Briefly, tissue was dissolved in TRIzol (Invitrogen), and 0.2 ml of chloroform was added. Samples were mixed thoroughly and centrifuged at 12,000 *g* for 15 min. The upper aqueous phase was transferred, and the RNA was precipitated by the addition of 0.5 ml of isopropanol followed by a 10-minute incubation at room temperature and centrifugation at 12,000 *g* for 10 min. The precipitate was washed with 75% ethanol, and the final pellet was resuspended in RNAse free water. To remove any traces of DNA, samples were treated with Turbo DNAse (Ambion, Austin, TX), for 2 hrs. All samples were tested to rule out DNA contamination.

OE RNA (2 µg) was reverse transcribed (RT) using Superscript II (Invitrogen). As a control, samples were also mock reverse transcribed (MRT, 1 µl of water added in lieu of 1 µl Superscript II). MRT reactions failed to produce PCR bands. As an additional control, primers were designed such that they spanned introns (see Table S1 for primer sequences). All PCR products were TOPO cloned (Invitrogen) and sequenced to confirm identity.

### Immunofluorescence

For the majority of the immunofluorescence experiments, sections were processed as described previously [22]. All washes were done gently and with special care in order to preserve tissue integrity. For the immunofluorescence of OMP and GAP-43, sections were thawed, air dried and then incubated with 2% bovine serum albumin (BSA) (Sigma, St. Louis, MO) in PBS-T (PBS with 0.3% Triton X-100, Sigma) for 30 min to block nonspecific binding sites. Incubation with primary antibodies diluted in blocking buffer was performed overnight at room temperature (RT). Sections were then washed 3 times in PBS-T for 5 min and incubated in secondary antibodies conjugated to Alexa Fluors (Molecular Probes, Eugene, OR). Sections were washed as above, rinsed in PBS, mounted in Gel/Mount mounting medium (Biomeda, Foster City, CA), and coverslipped. For immunodetection of PC1 and MKS3, previously described antibodies were used [21], [23]. G_olf_ and AC3 antibodies were obtained from Santa Cruz, α-acetylated tubulin antibody was obtained from Sigma, GAP-43 antibody was obtained from Novus Biological, OMP antibody was from Wako Laboratory Chemicals, and the generation of the MOR18-2 antibody was described above. MOR28 antibody was a generous gift of Dr. Richard Axel. NCS-1 antibody was clone #44162 [24]. DRAQ5 was purchased from Biostatus Ltd.

Free floating staining was done on olfactory epithelium peeled from mouse nasal septum after mice were perfusion fixed. The staining protocol followed the one described above and after the final wash tissue was mounted in between two coverslips.

Immunofluorescence for MKS3 and NCS1 was done using a slightly different protocol, because both primary antibodies were raised in rabbits. Briefly, after blocking only one of the primary antibodies was incubated overnight. Sections were washed and incubated with goat anti-rabbit F_AB_ fragments for 4 hs at room temperature. After washing, an Alexa Fluor donkey anti-goat secondary antibody was added for 1 h. Sections were washed, fixed in 4% PFA for 10 min and after several rinses, sections were subjected to a regular immuno protocol for the other antibody. Controls were done by replacing the second primary antibody by blocking buffer.

### Co-Immunoprecipitation

Co-immunoprecipitation experiments from transfected COS cells were performed using standard methods. Experimental details can be found in the SI.

### Electron Microscopy

The olfactory epithelium from the nasal septum was peeled, stained with osmium tetroxide and embedded for thin sectioning in EPON. Sections of 70–100 nm were examined on a JEOL transmission electron microscope and photographed at primary magnifications of 4,000–30,000X [25], [26].

## Results

### Multiple Renal Cystic Proteins are detected in the OE by RT-PCR

**Figure 1:**
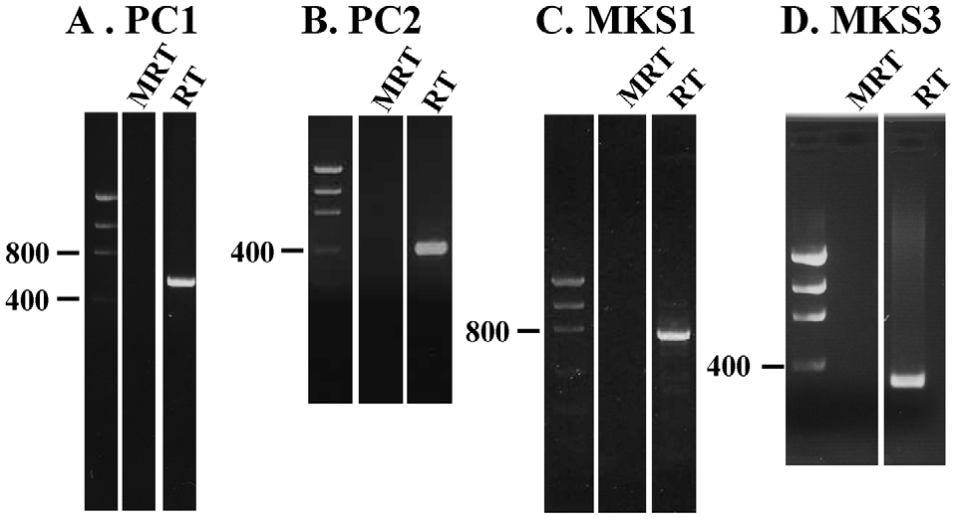
Proteins associated with renal cystic disease are expressed in the OE on the RNA level. Each panel shows a low DNA mass ladder (Invitrogen 10068-013) as well as a MRT (Mock RT negative control) and RT (experimental) lane. In addition to the MRT control, primers were designed such that introns in the genomic DNA would ensure that size of amplified genomic DNA would be appreciably different from the reverse transcribed band. Figure 1A shows the presence of PC1 in the OE (expected: 577 nt, genomic: 1627 nt), 1B shows PC2 in the OE (expected: 406 nt; genomic: 3447 nt), 1C shows MKS1 (expected: 715 nt, genomic: 4516 nt) and 1D shows MKS3 (expected: 309 nt, genomic: 7968 nt). All bands were cloned and sequenced to confirm identity.

We first demonstrated, using RT-PCR, that mRNAs encoding “renal cystic” proteins are expressed in the OE. Figure 1A–D demonstrates the expression of PC1, PC2, MKS1 and MKS3 transcripts in total RNA extracts from the OE. In all cases, appropriate controls were performed to demonstrate that no bands were detected when the OE RNA was not reverse transcribed. The resultant bands were cloned and sequenced, and found to be identical to previously published sequences (PC1: NM_013630.2, PC2: NM_008861.2, MKS1: NM_001039684.1, MKS3: NM_177861.3).

### MKS3 and PC1 localize to mature OSN by immunofluorescence

**Figure 2:**
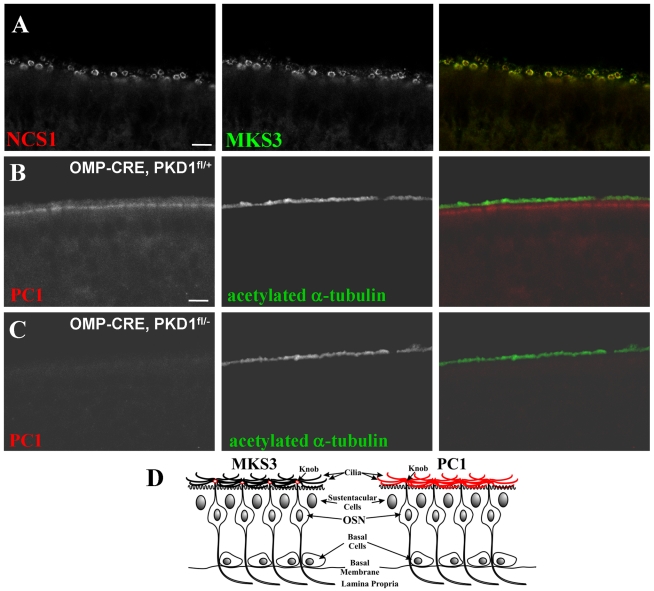
MKS3 and PC1 are localized to OSNs in mouse OE. Figure 2A demonstrates MKS3 localization in a pattern along the edge of the epithelium, a localization characteristic of proteins associated with dendritic knob membranes [27]. Colocalization with NCS1, previously shown to localize to OSN knobs [27], support this subcellular distribution. Figure 2B demonstrates the localization of PC1 to OSNs, along the edge of the nasal cavity. PC1 localization is primarily restricted to dendritic knobs and to cilia. Figure 2C demonstrates the lack of immunoreactivity of the PC1 antibody in an olfactory-specific PC1 null animal (*OMP-CRE, PKD1^flox/-^*). Because CRE expression in these animals is driven by OMP (which is only expressed in mature OSNs), the lack of staining in *OMP-CRE, PKD1^flox/-^* animals indicates that PC1 expression is restricted to mature OSNs. Figure 2D is a representation of the OE, showing the observed distribution pattern of MKS3 (left) and PC1 (right). Scale bars = 5 µm: in A; 10 µm: shown in (C) for C & D.

The olfactory epithelium encompasses multiple cell types, but only the mature OSNs play a direct role in mediating olfaction. To better characterize which cells are expressing these “renal cystic” proteins we next used antibodies directed against two of these proteins to determine their cellular and subcellular localizations. Figure 2A shows that MKS3 localizes to the dendritic knobs of OSNs. To confirm the localization of MKS3 we double-labeled for neuron specific calcium sensor 1 (NCS1; [27]), which in the OE is expressed exclusively in OSN knobs. As shown in Figure 2A, both markers showed the same pattern of expression (Figure S1). The specific localization of MKS3 to the dendritic knob was further confirmed by double-labeling with an antibody directed against α-acetylated tubulin, which labels OSN cilia. As shown in Figure S2, MKS3 is not expressed in the cilia as evidenced by the absence of any colocalization with α-acetylated tubulin. In Figure 2B PC1 within the OE is shown localizing o the edge of the nasal cavity. The distribution of PC1 is more diffuse than that of MKS3, however, suggesting that it is present in both the dendritic knobs and in the cilia (Figure 2D). Figure 2C demonstrates that the PC1 antibody is unable to detect signal in a mouse model in which PC1 expression has been selectively deleted in the mature OSNs (using *OMP-cre, PKD1^flox/-^* mice). These data indicate the specificity of the antibody, and demonstrate that PC1 localizes exclusively to mature OSNs. The *OMP-cre, PKD1^flox/-^* mice showed no differences in the expression or localization of other OE proteins when compared with control (see Figure S3).

### Expression of OE proteins in rats with a disease-causing MKS3 mutation

**Figure 3:**
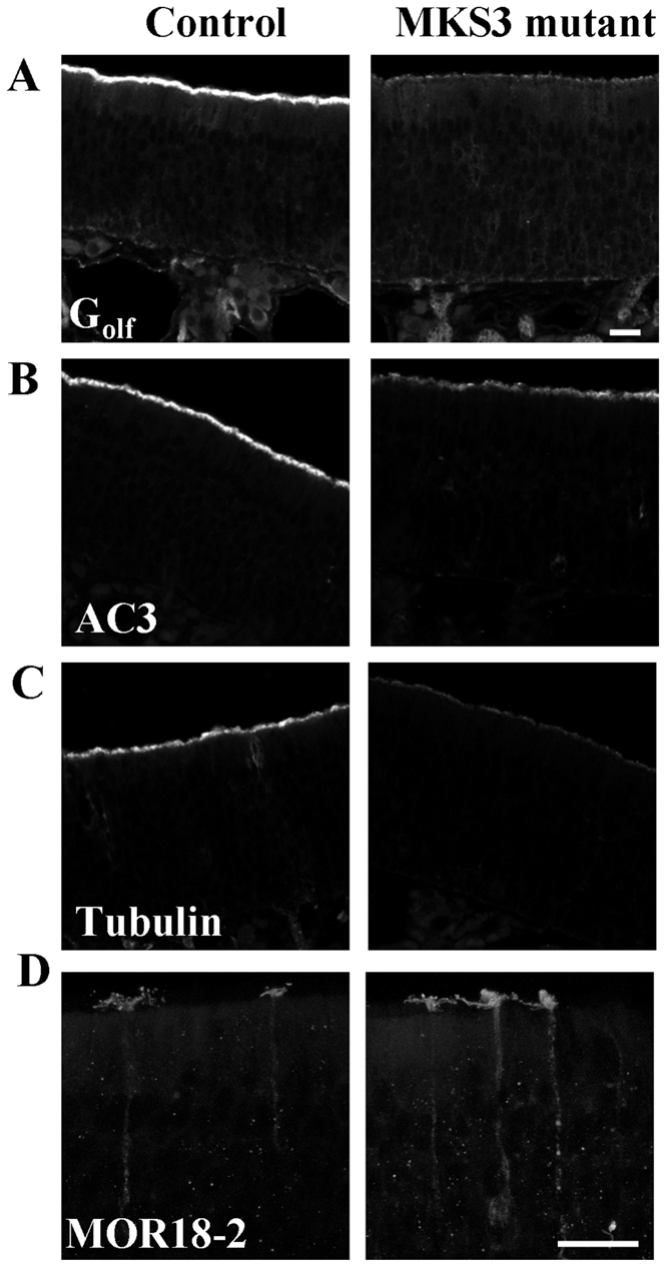
Expression and localization of olfactory proteins in the OE of *MKS3* mutant rats. Representative pictures demonstrating that rats homozygous for a point mutation in *MKS3* have decreased G_olf_ (A), AC3 (B), and α-acetylated tubulin (C; a ciliary marker) expression as compared to control rats. MOR18-2, however, appears to be expressed normally (shown in (D) as a compressed z-stack). Scale bar = 20 µm: shown in (A) for A–C, and in (D) for D.

We next took advantage of a previously published rat model harboring a disease-causing point mutation in the gene encoding MKS3 [18], which results in polycystic kidney disease [17] as well as hydrocephalus [9]. We performed a series of immunofluorescence experiments to examine the expression and distribution of several key OE proteins in these animals. All immunofluorescence experiments were performed on four *MKS3* mutant rats as well as five control rats. *MKS3* mutant rats had a dramatic decrease in G_olf_ expression as compared to control animals (Figure 3A). Likewise, we found that the expression of AC3 was reduced in *MKS3* mutant rats (Figure 3B). Both are proteins associated with the primary sensory cilia in OSNs. We further examined ciliary proteins with immunofluorescence staining assessing the distribution of the general ciliary marker α-acetylated tubulin. Similar to G_olf_ and AC3, the expression of α-acetylated tubulin was reduced (Figure 3C; additional images that document the reproducibility of this pattern between animals are shown in Figure S4a–b). We next wished to determine whether OR expression is altered in *MKS3* mutant rats. Because we were unable to find antibodies that recognized ORs in rat OE, we developed a new antibody against MOR18-2. The sequence of the peptide used to generate this antibody is 100% identical in mouse (MOR18-2) and in rat (Olr59). The characterization of this new antibody is shown in Figure S5. Olr59 expression exhibited a similar staining pattern in *MKS3* mutant and in control rats. Olr59 positive OSNs were observed in zone 1 (dorsal zone) in the OE, as expected for this class I OR, and was present throughout the cell, including the OSN cilia, dendritic knob, cell body, and axon (Figure 3D). Of interest, in *MKS3* mutant rats the dendritic knobs of the Olr59-positive cells appeared swollen, and the number of cilia per knob appeared to be reduced (see below).

**Figure 4:**
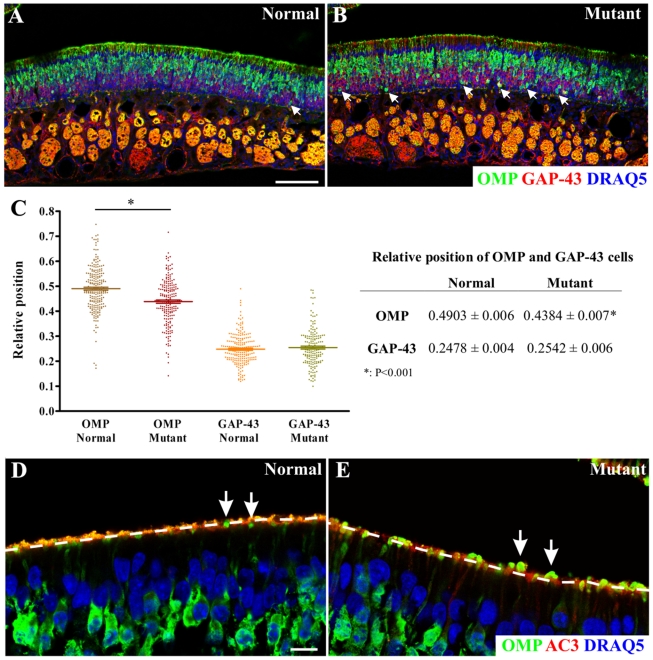
Altered distribution of OMP cells and abnormal dendritic knobs are observed in rats with a disease-causing point mutation in *MKS3*. *MKS3* mutant (B) sections stained with OMP (green) and GAP-43 (red) showed high numbers of OMP^+^ cells at the bottom of the OE (arrows) as compared to Control (A; nuclei are stained with DRAQ5 (blue)). C: Quantification of the relative position of OMP and GAP-43 expressing cells confirmed the unusual basally-oriented distribution of OMP cells in *MKS3* mutant rats (ANOVA P<0.0001, Bonferroni post test values are shown in the table). There was no significant change in the relative position of GAP43 cells between genotypes. At a low magnification (as in A, B), the edge of the epithelium appeared to be continuous in the control animals (as shown by OMP staining), but had a discrete, interrupted staining pattern in the mutant animals. At higher magnification (D, E), this appears to be due to an increased frequency of OMP knobs (green) appearing to protrude “above” the ciliary layer (as defined by AC3 (red) staining, dashed line; nuclei are stained with DRAQ5 (blue)). Notice that OSN knobs appeared to be bigger in the mutant animals (arrows in D and E). Scale bars  = 100 µm: shown in (A) for A & B; 10 µm: shown in (D) for D & E.

We then stained the OE from both control and *MKS3* mutant rats with markers of the different OE cell types in order to determine if the basic tissue organization and stratification were intact. Staining for GAP-43 (a marker of immature OSNs) and OMP (a marker for mature OSNs) revealed a disruption in the organization of the MKS3 mutant OE. As shown in Figure 4 (arrows), many spherical OMP^+^ cells were located at the bottom of the OE, where normally basal stem cells and GAP-43 expressing cells are found. (Figure 4A, B). To quantify this irregular distribution, we examined along the septum the nuclei of each OMP^+^ or GAP-43^+^ cell and scored them for location in the OE. The surface of the OE, at the level of the lumen, was scored as “1”, and the base of the epithelium at the level of the basal lamina, was scored as “0”. In the *MKS3* mutant rats, the OMP^+^ cells were significantly more basal in their distribution relative to controls, although there was no difference between genotypes in the location of GAP-43^+^ cells (Figure 4C). At low magnification (as in Figure 4A, B) it was also noted that the OMP staining at the edge of the epithelium was composed of discrete separated puncta in the *MKS3* mutant animals while controls had a more uniform and uninterrupted expression of OMP at the luminal surface of the OE. Of interest, high magnification images showed a fundamental change in the organization of the *MKS3* mutant OE. In controls, OMP^+^ dendritic knobs end at the surface of the OE, with the AC3^+^ cilia extending above the knobs and into the lumen (Figure 4) However, in the *MKS3* mutants the OMP^+^ dendritic knobs often extended above the level of the AC3^+^ cilia (Figure 4D, dashed line). Moreover, some of the OSN dendritic knobs in the *MKS3* mutants appear larger than those in controls (Figure 4D, E, arrows). A similar phenotype is also found following mutation of the intracellular trafficking protein PACS-1, which is also expressed in OSN knobs [28]. These abnormalities are consistent with a failure of the OE to properly organize in animals harboring mutations in genes associated with cystic diseases.

### Electron microscopy of the olfactory epithelium in rats with a disease-causing MKS3 mutation

**Figure 5:**
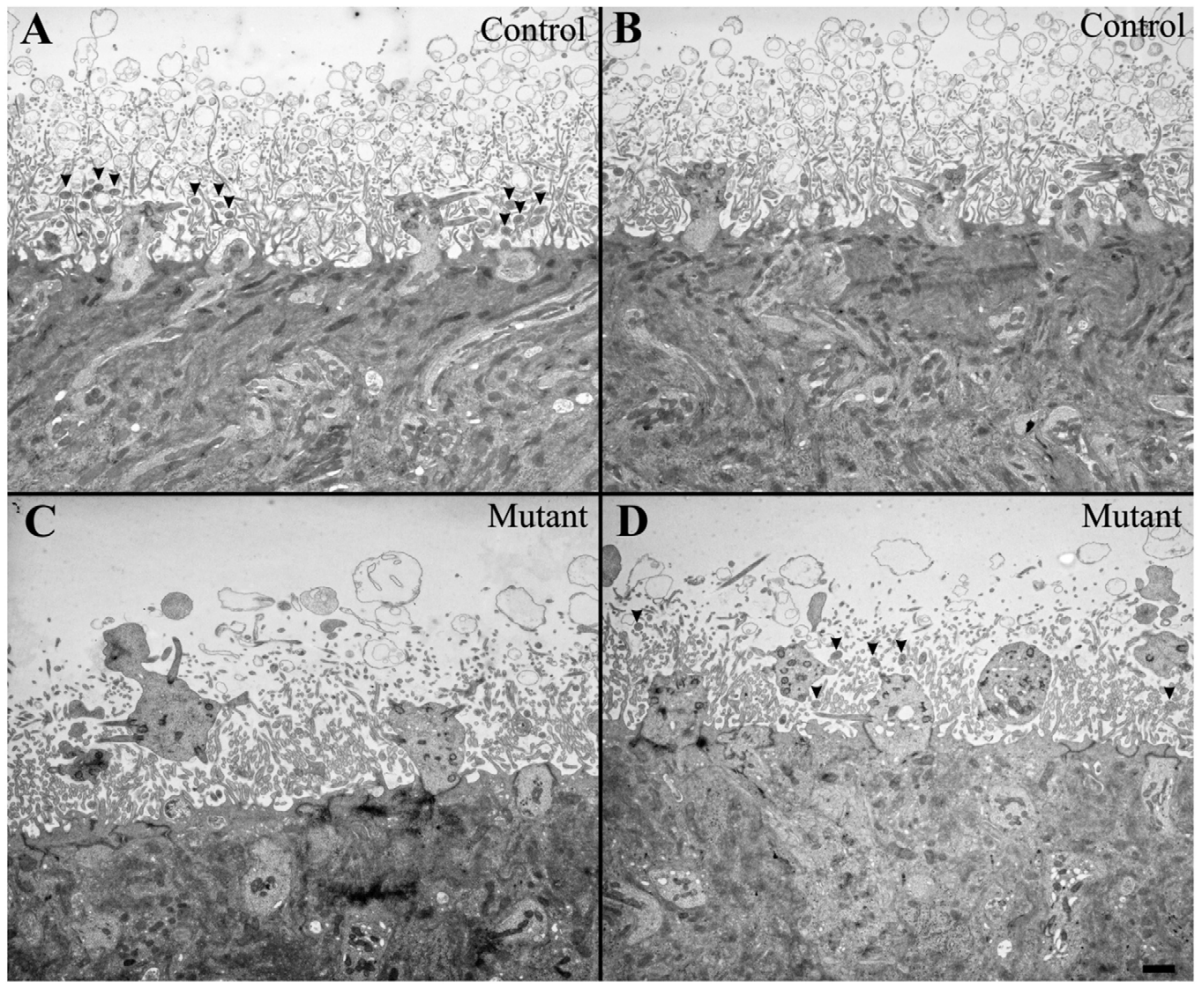
Swollen knobs and a decreased number of cilia are detected in mutant *MKS3* mutant rat olfactory epithelium. Representative olfactory epithelium images from normal (A, B) and MKS3 mutant (C, D) rats. Mutant sections showed enlarged OSN knobs, some of which exhibited irregular shapes (e.g., to the left in C), with knobs frequently appearing to be “detached” from the OE surface. At this magnification, coronally sectioned OSN cilia could be distinguished from microvilli as a result of their different sizes. Control sections showed multiple easily identifiable cilia (arrowheads, only shown in A), while mutant sections showed a decreased number (arrowheads in D; none could be recognized in C). Scale bar = 1 µm: shown in (D) for A–D.

**Figure 6:**
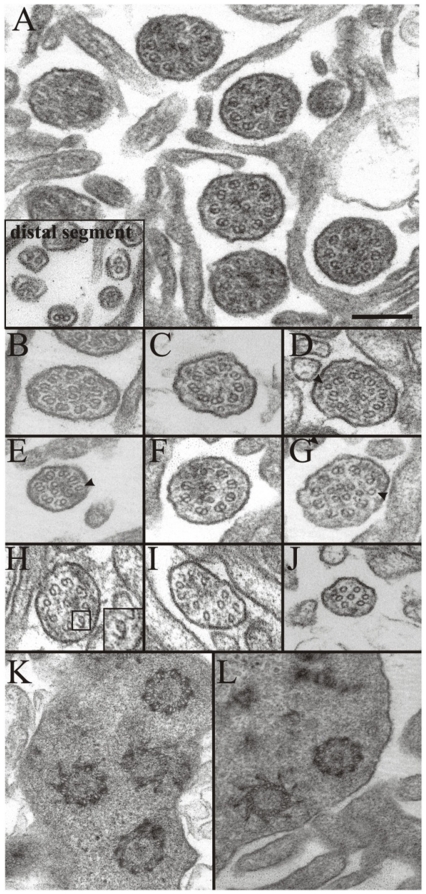
Mutant cilia showed altered microtubule organization. A: Representative group of cilia in the control sections with the characteristic 9+2 organization of microtubules. The insert shows the distal segment of the cilia. B–J: Cilia from *MKS3* mutant rats showing altered microtubular organization. B: 9+2; C: 9+0; D: (9+1)+2, an extra singlet in the periphery (arrowhead); E: (7+1)+2; F: 8+4; G: (9+1)+6; H: (7+1)+1; I: 11; J: 7. In some cases the external doublet showed the circumference of one of the microtubules to be discontinuous or “opened” (F, H insert). No differences could be detected in basal bodies from normal (K) or mutant (L) animals. Scale bar  = 200 nm: shown in (D) for A–L.

To further characterize these anomalies, we used electron microscopy to study the ultrastructure of the OE. Figure 5 shows the general structure of the surface of the OE in control (top) and *MKS3* mutant (bottom) animals. In agreement with our confocal observations, cystic animals showed dendritic knobs that were swollen with abnormal shapes (e.g. blebs protruding from the knob); in many cases the knobs extended far into the lumen. At the ultrastructural level we identified coronally sectioned OSN cilia in the control animals (Figure 5, filled arrowheads top left) but it was difficult to recognize them in the *MKS3* mutants (Figure 5, filled arrowheads bottom right). When we turned our attention to individual cilium, ultrastructural differences were pronounced. Cilium from control rats (Figure 6A) showed the typical 9+2 ultrastructural organization of microtubules, characteristic of the proximal segment of OSN cilia. Cilia with two singlet microtubules (Figure 6A insert) were also seen, characteristic of distal segments of OSN cilia (reviewed in [29]). In *MKS3* mutant rats, however, cilia with the normal 9+2 microtubule structure were rarely observed (Figure 6B). More commonly, the cilia from *MKS3* mutant rats showed a severe perturbation of microtubule organization (Figure 6C–J). In the *MKS3* mutants we observed an absence (Figure 6C) or increased number (Figure 6F, G) of singlet microtubules in the center of the cilia; singlets in the periphery of cilia (Figure 6D, E, G, H); doublets in which one of the microtubules is open (Figure 6F, H); or microtubules without any evident organization (Figure 6I, J). However, when we turned our attention to the basal bodies in the dendritic knobs, no significant differences were detected between the two genotypes (Figure 6K, L). All these data point to MKS3 playing an important role in organizing the structure of OSN cilia.

### Expression of OE proteins in mice with a mutation in MKS1

**Figure 7:**
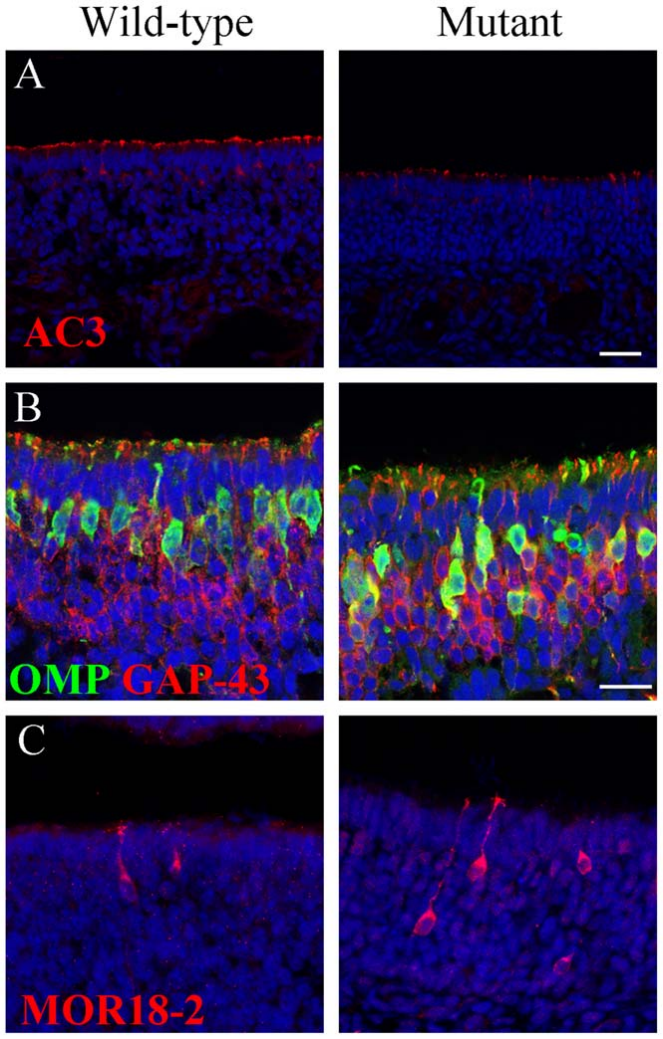
Expression and localization of olfactory proteins in the OE of *MKS1* mutant mice. Mice homozygous for a mutation in *MKS1* have (A) decreased AC3 in the OE compared to wild-type littermates. *MKS1* mutant mice also exhibit colocalization of OMP and GAP-43 to a greater extent than in wild-type mice (B), despite the apparently normal localization and expression of MOR18-2 (C). Scale bars are 50 µm (shown in (A)), and 20 µm shown in (B) for (B) and (C); nuclei are stained with DRAQ5 (blue).

Finally, we examined the OE of a recently-described *MKS1* mutant mouse [10]. Because this mutation results in an embryonic lethal phenotype, we examined the OE of pups at E18.5. In mice homozygous for the *MKS1* mutation, there is a decrease in the expression of AC3 in the cilia relative to wild-type (Figure 7). To further assess cellular organization of the OE we stained the OE of *MKS1* mutant mice with OMP and GAP-43, as markers of OSN maturation. We observed that OSNs colocalizing both OMP and GAP43 were more frequent in *MKS1* mutant mice than in controls. We then asked if the expression of two different ORs in the OE was affected by the *MKS1* mutation. Both MOR18-2 (Figure 7C), and MOR28 (Figure S6), showed patterns of expression in OE zones 1 and zone 4, respectively, that did not differ between the mutant mice and controls (the MOR28 antibody was not used in the MKS3 studies detailed above because it does not recognize the rat orthologue of the MOR28 protein). Both proteins also had a normal subcellular distribution in OSNs.

In summary, our findings for MKS1 and MKS3 support the hypothesis that proteins associated with renal cystic disease play important roles in modulating the proper expression and/or localization of a subset of OE ciliary proteins, and in the maturation of OSNs.

## Discussion

We report that multiple proteins implicated in renal cystic disease are expressed in the OE, where they localize to OSNs. We found that multiple renal cystic proteins are present in the OE on the mRNA level (MKS1, MKS3, PC1, PC2), and that PC1 and MKS3 proteins localize chiefly to the apical compartments of OSNs. PC1 is found in the cilia and the dendritic knobs, whereas MKS3 is restricted to the dendritic knobs. Additionally, we found that rats with a disease-causing mutation in *MKS3* have reduced expression of key olfactory ciliary proteins (AC3, G_olf_). Interestingly, OSN knobs are swollen and cilia structure is severely disrupted in MKS3 mutants. Furthermore, the pseudostratified organization of the OE itself (as delineated by staining for OMP and GAP43) is disrupted in the *MKS3* and *MKS1* mutants. In the *MKS1* mutant mice we also found decreased expression of a key olfactory protein (AC3). However, OR expression appears normal in both *MKS3* and *MKS1* mutants, where they localize to the cilia despite the failure of ciliary localization of AC3, G_olf_, or α-acetylated tubulin. This result, taken together with the survival of most of the MKS3 mutant pups after birth, suggests that these animals must have some ability to smell, since severe olfactory deficits typically result in neonatal death due to failure of olfactory cues to initiate suckling. Although more subtle differences in olfactory sensitivity and/or discrimination cannot be ruled out, this is beyond the scope of the present work. Overall, these data indicate that renal cystic proteins (known to be associated with ciliopathies, and with cilia and basal bodies in the kidney) co-localize to similar structures in OSNs in the OE, and that mutations in these proteins are associated with perturbation of OSN organization and defects in ciliary structure. It will be interesting in the future to analyze the embryonic progression of disease in these animal models, in order to determine whether the normal progression of OSN development is also altered.

Previous studies identified “renal” proteins that are associated with the OE (NPHP6 [15], and BBS proteins [16]). Furthermore, these studies suggested that renal-disease-causing mutations in these proteins (which are associated with both Joubert and Meckel syndromes, and Bardet-Biedl Syndrome, respectively) can also result in anosmia. It is now apparent that future studies examining patient populations with classic forms of renal cystic disease – such as ARPKD (autosomal recessive PKD) – are warranted to determine if comorbidity for olfactory-related sequelae occurs.

Our finding that both PC1 and PC2 are expressed in the OE, along with the MKS1, MKS3, and BBS proteins [16] and NPHP6 [15] demonstrate that renal cystic proteins are likely to play a key role in the function of the OE, although their exact roles in the OE are as yet unclear. The renal cystic proteins whose subcellular expression we and others have examined (NPHP6, MKS3, PC1) all localize to the OSN dendritic knob. Colocalization of these proteins is consistent with the possibility that renal cystic proteins may form an interactive complex. It is tempting to speculate that renal cystic proteins in the dendritic knob may play a role in regulating protein trafficking into and out of the cilia and/or in regulating proper microtubule organization within cilia. Indeed, in the OE of *MKS3* mutant rats we found that several ciliary proteins failed to localize properly, and that the cilia themselves were malformed. Consistent with this observation, in renal cystic diseases improperly formed cilia are a common feature in the kidney. Therefore, it is plausible that ciliary trafficking defects, and/or defects in cilia formation, contribute to the pathogenesis of these diseases in both the OE and in the kidney.

Proper ciliary localization of characteristic OSN proteins is altered when BBS or NPHP6 proteins are mutated (9, 10). Similarly, we observed a reduction in the expression of OSN ciliary markers after the mutation of MKS proteins. Moreover, a point mutation in *MKS3* was sufficient to significantly affect ciliary microtubule organization and to reduce the number of properly formed cilia. Intriguingly, *MKS3* mutant renal epithelial cells have frequently form >1 cilia (as opposed to the single primary cilium usually found in the kidney) and appear longer than those in wild-type controls [1]. The different ciliary phenotypes found in the kidney and the nose of *MKS3* mutant animals may reflect a different role of MKS3 in regulating cilia formation in cells with a single, primary cilium (renal epithelial cells) as opposed to cells with multiple cilia (OSNs). To our knowledge, transmission electron microscopy has not yet been performed on MKS3 mutant renal cilia, so it is unknown whether renal cilia have perturbations in microtubule doublet number or arrangement.

Although it could be argued that the reduction in AC3 and G_olf_ observed in the present study is due solely to the decreased number of cilia in the OE, we believe it is more likely that an alteration in transport of proteins into the cilia also plays a role. The clear ciliary localization of ORs in the *MKS1* and *MKS3*mutant animals implies that a reduction in cilia number alone cannot explain the decrease in AC3 or G_olf_ staining. Indeed, the localization of MKS3 to the OSN knobs places this protein in the perfect position to be participating in ciliary trafficking/organization.

Of particular interest is the disrupted pseudostratified organization of the OE in the MKS3 mutant rats, as shown in Figure 4. A similar finding was also shown for the *MKS1* mutant mice (Figure 7b). These findings, together with the aberrant tendency for swollen knobs to protrude above the ciliary layer in the *MKS3* mutants, indicate a defect in the maturation process of OSNs, and consequently a flaw in either the initial organization, or in the maintenance, of the OE. These data, coupled with the localization of MKS3 to the dendritic knob, suggests that proper function of the dendritic knob is necessary in order to promote proper cellular organization. Although we do not yet know the mechanism underlying these changes, MKS3 has topological homology to Frizzled receptors (transmembrane receptors for the Wnt family of intercellular signaling molecules). Wnts have been implicated in, among other functions, cell type specification and polarization. It has been suggested that one of the likely ligands for MKS3 is Wnt-5a [30], a Wnt molecule that is thought to participate primarily in the non-canonical Wnt signaling pathway, which regulates planar cell polarization. Intriguingly, Wnt-5a has been shown to play a key role in the development and organization of the olfactory epithelium [31]. In addition, errors in planar cell polarity orientation are thought to contribute to the pathogenesis of at least some of the renal cystic disease [32], and it has been suggested that dysregulation of Wnt signaling plays an important role in this process [33]. It is tempting, therefore, to speculate that some of the alterations observed in MKS3 mutants may be due to alterations in the Wnt signaling pathway.

As a first approach to elucidate the potential role of MKS proteins, we performed co-immunoprecipitations with ORs and either MKS1 or MKS3, using COS cells transfected with a cDNA encoding full-length human MKS3 (or MKS1-HA) as well as with cDNA constructs encoding ORs modified through the addition of an N-terminal Flag tag (Figure S7). We found that MKS3 and MKS1 can interact with ORs. All five ORs tested pulled down both MKS3 and MKS1, and although the efficiency of the pull-down varied, MOR18-2 and MOR256-25 appeared to have the strongest interaction with both MKS3 and MKS1. The five ORs chosen for this study included one “classical” OE OR (MOR EG), as well as 4 ORs which we previously reported are expressed in renal tissue [22]. Although the significance of this potential for interaction is not yet known, it is intriguing that MKS3 has a membrane topology similar to that of ORs (7 transmembrane domains, with an extracellular N-terminus). With the exception of the presence of a B9 domain, little is known about the structure of MKS1 [23]. In view of these data showing that MKS1 and MKS3 can interact with several ORs (including MOR18-2), and that MOR28 and MKS3 colocalize in the knobs (Figure S8), it is of interest that MOR18-2 expression in *MKS3* mutant rats and MOR18-2 (and MOR28) expression in *MKS1* mutant mice were normal. This altered expression of (non-OR) ciliary proteins, the disorganized cilia ultrastructure and the apparently normal expression of MOR18-2 in OSN cilia suggests either that: (a) the *MKS3* and *MKS1* mutations in these models do not disrupt the MKS-OR interaction; or (b) that the putative MKS-OR interaction does not regulate OR trafficking. Although it is clear that the MKS proteins interact with multiple ORs *in vitro* with different affinities, the extent and role of interactions between MKS1 and MKS3 and different ORs in the OE *in vivo* remains to be fully elucidated.

In conclusion, we have demonstrated that multiple genes associated with renal cystic diseases are also expressed in the OE, where they localize to the OSN dendritic knob. Our data lead to the suggestion that these proteins play a role in the OE (and potentially in the kidney as well) to regulate proper ciliary function. We hope that better understanding of the role of renal cystic proteins in the OE will help reveal olfactory phenotypes in renal cystic diseases, such as that found by McEwen et al. [15]. Indeed, olfactory testing may eventually be useful as a non-invasive index for the presence, or progression, of ciliopathies. Understanding these interactions will give us important insights into the physiological roles of these proteins. How these proteins interact in OSNs, in a non-renal context, may provide new insights which can inform our understanding of renal cystic disease.

## Supporting Information

Figure S1 Control experiments are shown for double-staining with two rabbit antibodies, MKS3 and NCS1. MKS3 (A, C) or NCS1 (B, D) were used as the first primary antibody following the protocol described in the Methods. Control experiments were done by replacing the second primary antibody (NCS1 in C or MKS3 in D) by blocking buffer. Scale bar  = 10 µm.
(TIF)

Figure S2 Free-floating immunoflourescence of the OE, showing localization of MKS3 to the dendritic knobs. Many, but not all, knobs were positive for MKS3 (red, some of them marked with the arrows). α-acetylated tubulin (green) is also stained to show cilia. Inset: A higher magnification of the same field (dashed square), showing individual cilia (green) protruding from an MKS-positive knob. Scale bar  = 10 µm.
(TIF)

Figure S3 Olfactory epithelium expression and localization of various proteins is largely unaffected in mice null for PC1 in the OE. A. In mice null for PC1 in the OE (OMP-CRE, PKD1^flox/-^), the localization of AC3, G_olf_ and MKS3 are not affected (although, in some mice, the level of expression of MKS3 appears to be somewhat reduced). B. In addition, mOR28 (green; blue nuclei) and M50 (red; blue nuclei) properly localize and cilia appear normal.
(TIF)

Figure S4 There is a consistent decrease in the level of expression of Golf, AC3, and α-acetylated tubulin in the OE of MKS3 mutant rats versus controls (one picture shown per animal; n = 5 control, n = 4 mutant).
(TIF)

Figure S5 MOR18-2 antibody recognizes MOR18-2 protein *in vitro* and *in vivo*. A. Western blot of HEK 293T cells overexpressing various OR constructs. A band of the expected size (37 kDa), as well as other minor bands, were found only in cells overexpressing MOR18-2. B. Immunocytochemistry in HEK 293T cells using OR constructs containing an N-terminal Flag tag. Cells were transfected with MOR256-21 or MOR18-2 (as well as 256-25, 256-24, and EG – not shown). The MOR18-2 antibody specifically recognized MOR18-2, as shown by the colocalization of the MOR18-2 and monoFlag antibody signals. C. MOR18-2 recognizes zone 1 OSNs in MOR18-2^+/+^, but not MOR18-2^−/−^ mice (Scale bar = 20 µm; compressed z-stacks). Although this antibody gives a specific signal in the OE, in other tissues tested it cross-reacts with an unknown protein (as evidenced by identical antibody staining patterns in wild-type and null mice).
(TIF)

Figure S6 MOR28 zonal distribution is normal in MKS1 mutant mice. MOR28 staining is in red; nuclei shown in blue.
(TIF)

Figure S7 MKS3 and MKS1 both co-immunoprecipitate with OR constructs (molecular weight markers are indicated to the left of each blot). Flag-tagged OR constructs were co-transfected into COS cells along with MKS3, or HA-tagged MKS1. Lysates and unbound fractions are shown in Figure S6A (blotted with MKS3). For Figure S6B, immunoprecipitation was performed using a Flag antibody, and membranes were then blotted for MKS3. Co-expression of Flag-tagged MOR18-2, 256-21, 256-25, 256-24, and EG were all capable of facilitating the pull-down of MKS3, although the strongest signal was observed using MOR18-2 and 256-25. Figure S6C shows MKS1 lysates in the presence of various ORs, whereas Figure S6D shows the results of co-immunoprecipitation using a Flag antibody, followed by blotting for HA (MKS1). MKS1 also interacts with all of the ORs tested, with the strongest signal observed using MOR18-2, MOR256-25 and 256-24, and the weakest signal observed with MOR256-21.
(TIF)

Figure S8 MOR28 and MKS3 colocalize in dendritic knobs. Knobs expressing MOR28 and MKS3 (open arrows), as well as knobs expressing MKS3 alone (filled arrows) were observed. This suggests that in OSNs, ORs and MKS3 are expressed in the same compartment. Red is MOR28; Green is MKS3; Blue is DRAQ5. The square in the left is shown at higher magnification in the right. Scale bar = 20 µm (left), 2 µm (right).
(TIF)

Table S1 Primer sequences for both RT-PCR and for genotyping are shown.
(TIF)

Text S1 (PDF)
