# Supplementary figures and images for: Renal Cystic Disease Proteins Play Critical Roles in the Organization of the Olfactory Epithelium
Source: PLoS One. 2011 May 13;6(5):e19694. doi: 10.1371/journal.pone.0019694 (PMC3094399; doi:10.1371/journal.pone.0019694)

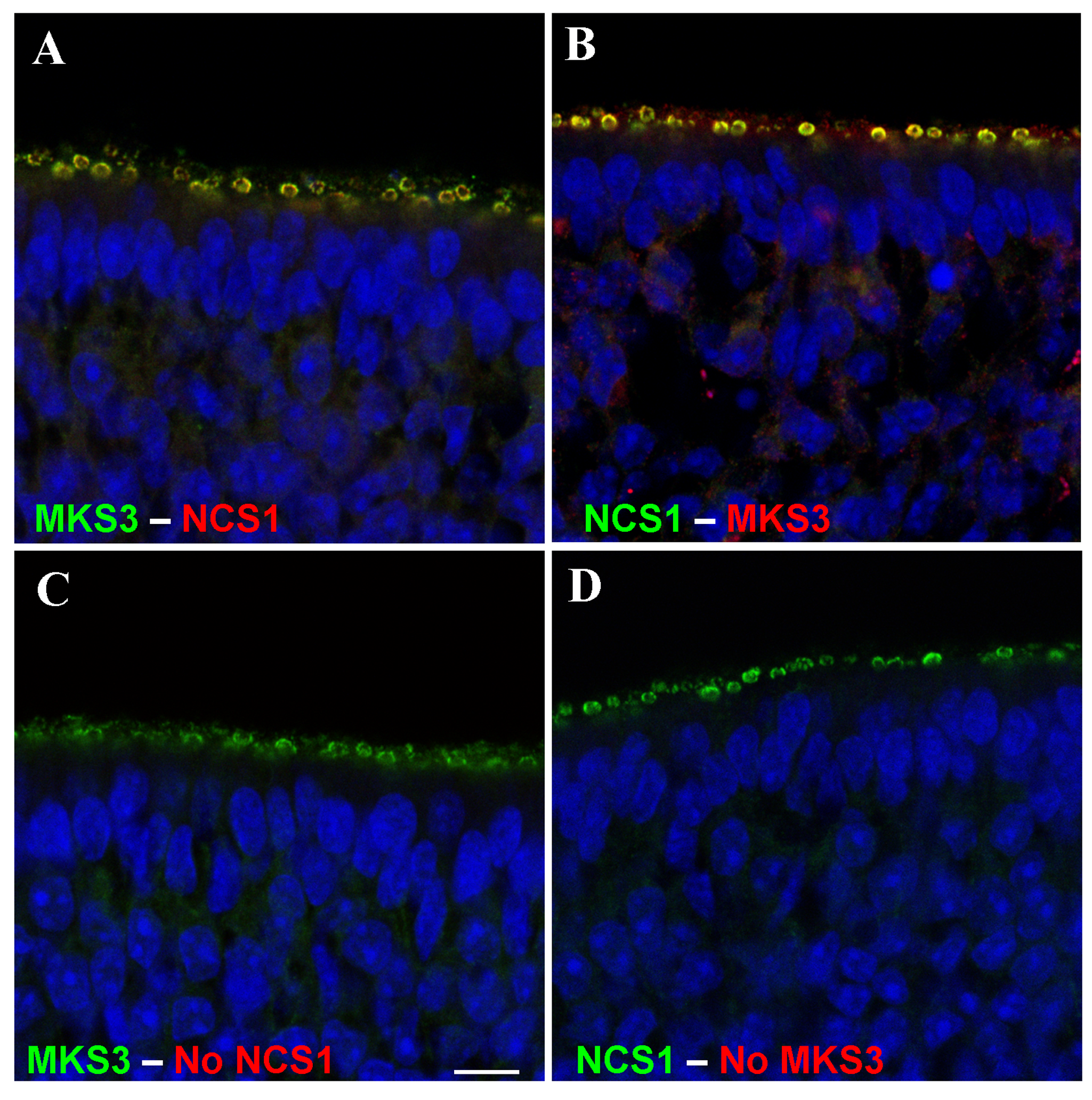

Supplement: Figure S1 — Control experiments are shown for double-staining with two rabbit antibodies, MKS3 and NCS1. MKS3 (A, C) or NCS1 (B, D) were used as the first primary antibody following the protocol described in the Methods. Control experiments were done by replacing the second primary antibody (NCS1 in C or MKS3 in D) by blocking buffer. Scale bar = 10 µm. (TIF) [file pone.0019694.s001.tif]

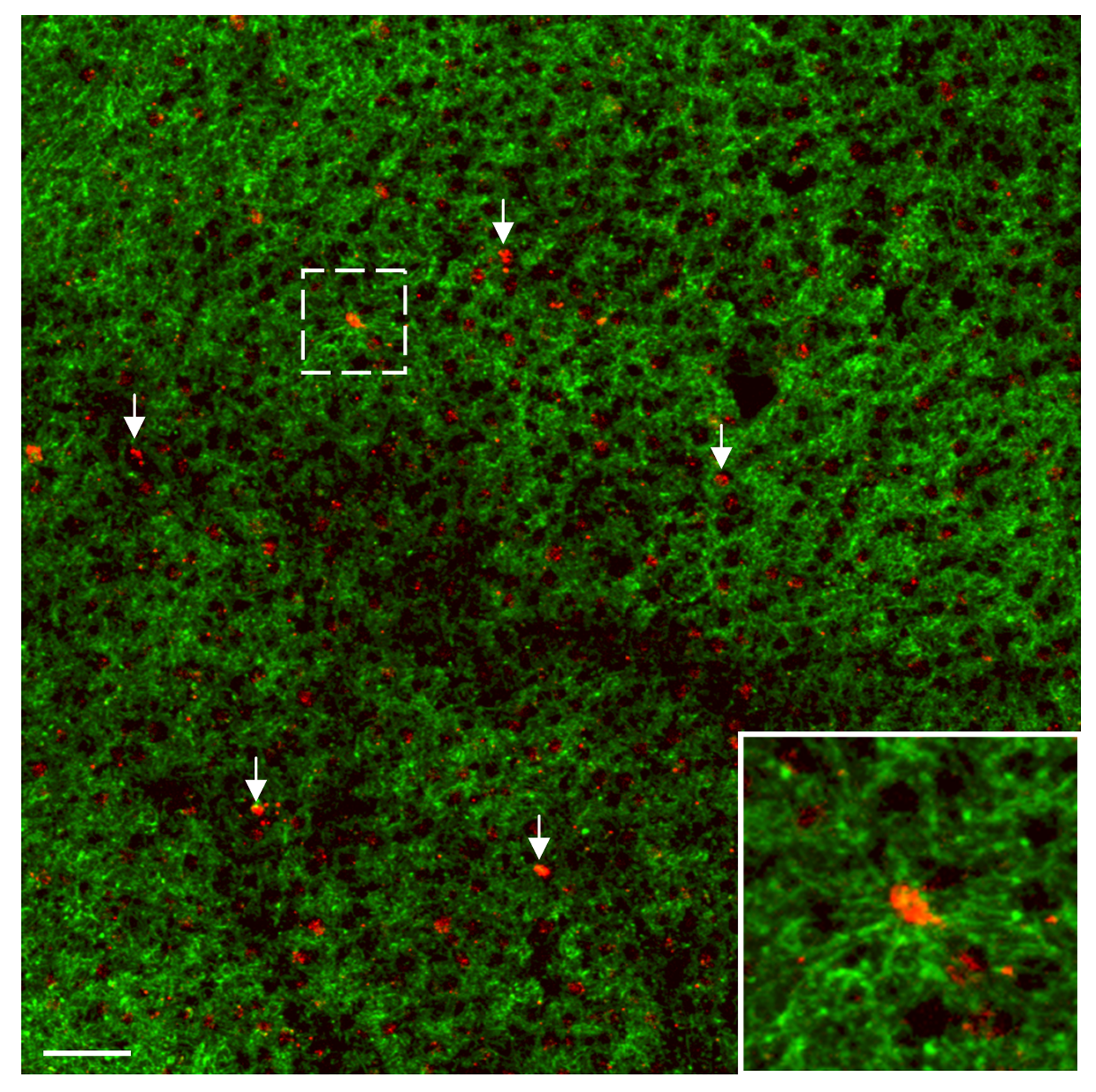

Supplement: Figure S2 — Free-floating immunoflourescence of the OE, showing localization of MKS3 to the dendritic knobs. Many, but not all, knobs were positive for MKS3 (red, some of them marked with the arrows). α-acetylated tubulin (green) is also stained to show cilia. Inset: A higher magnification of the same field (dashed square), showing individual cilia (green) protruding from an MKS-positive knob. Scale bar = 10 µm. (TIF) [file pone.0019694.s002.tif]

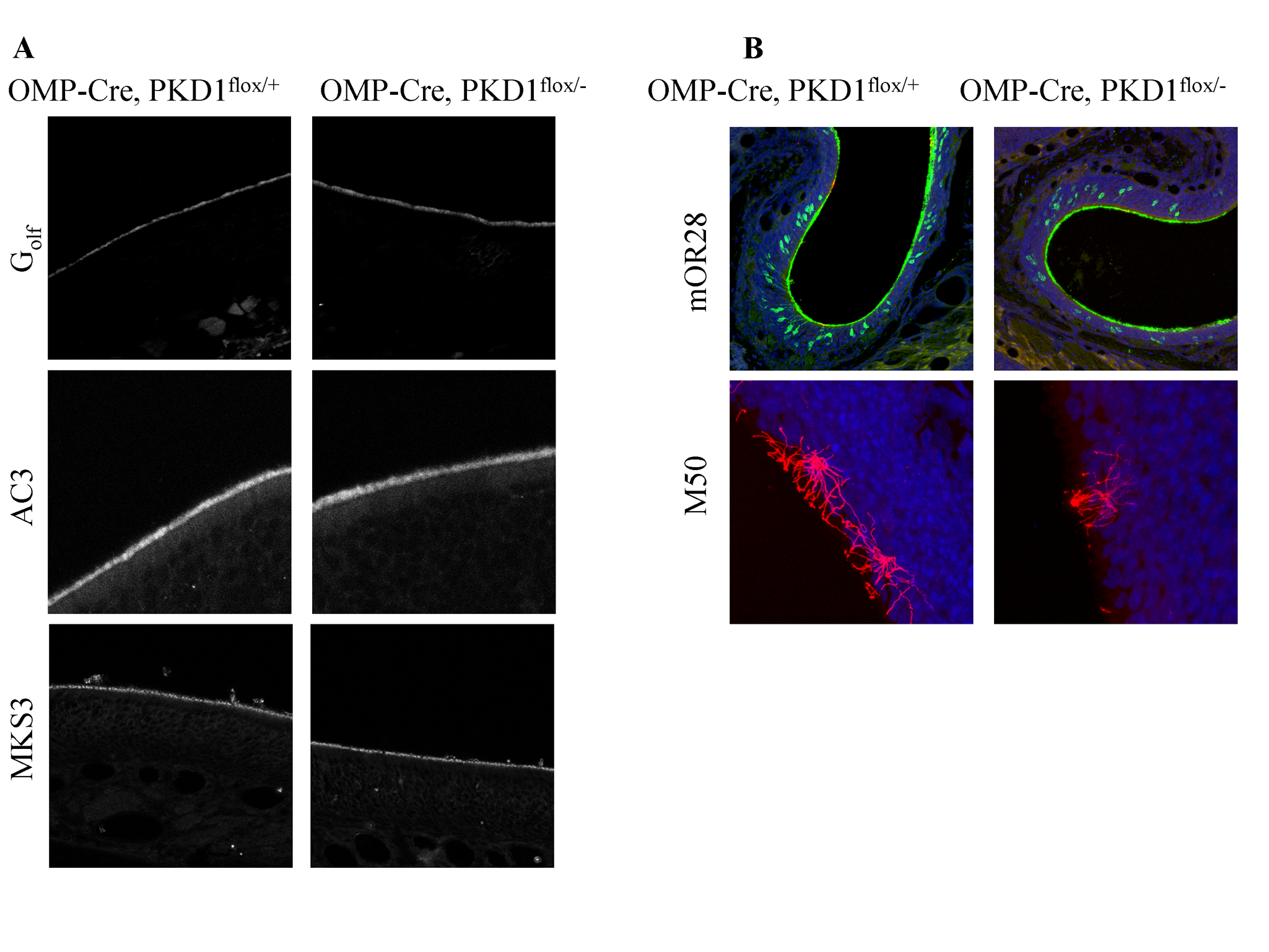

Supplement: Figure S3 — Olfactory epithelium expression and localization of various proteins is largely unaffected in mice null for PC1 in the OE. A. In mice null for PC1 in the OE (OMP-CRE, PKD1flox/-), the localization of AC3, Golf and MKS3 are not affected (although, in some mice, the level of expression of MKS3 appears to be somewhat reduced). B. In addition, mOR28 (green; blue nuclei) and M50 (red; blue nuclei) properly localize and cilia appear normal. (TIF) [file pone.0019694.s003.tif]

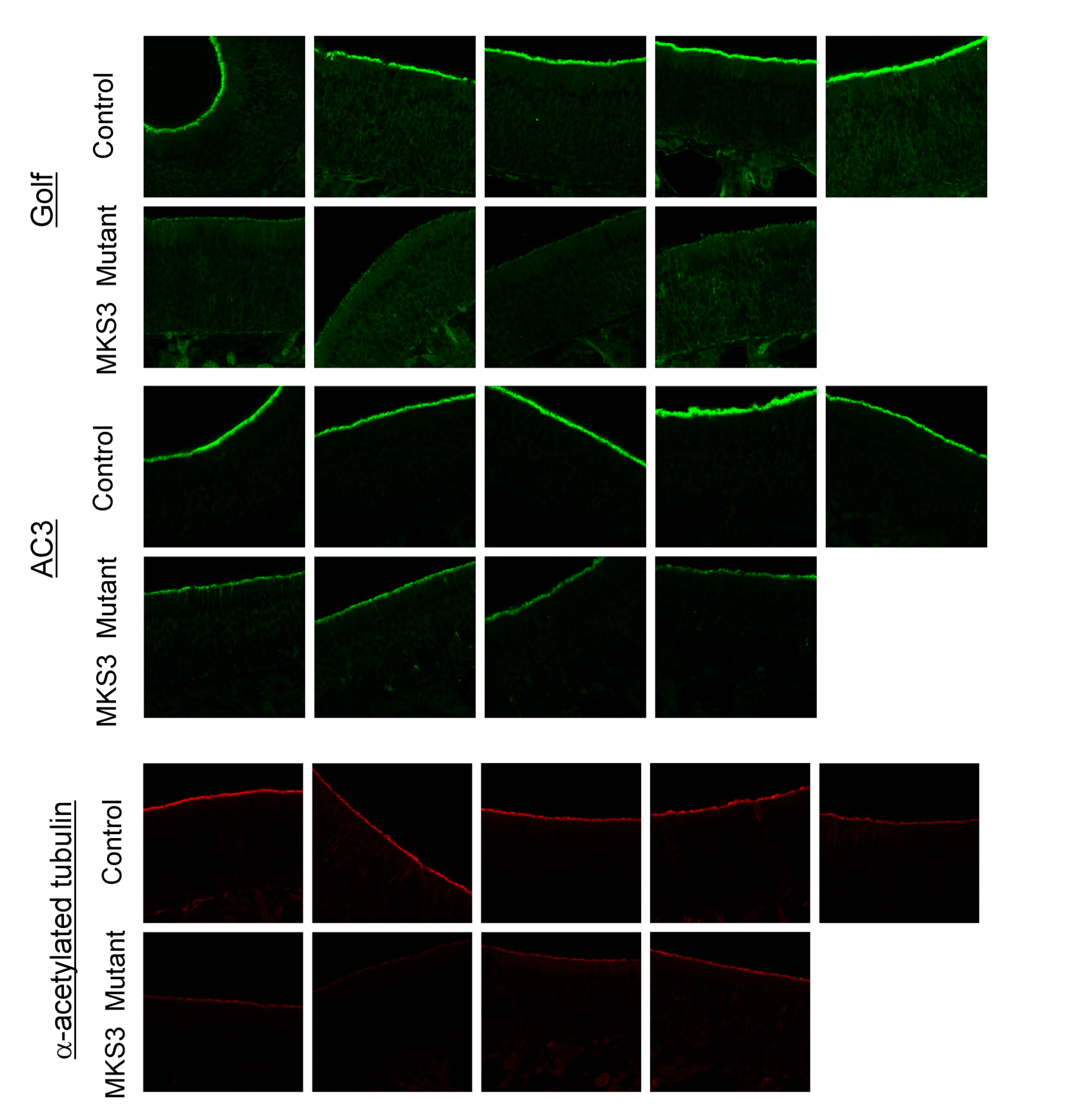

Supplement: Figure S4 — There is a consistent decrease in the level of expression of Golf, AC3, and α-acetylated tubulin in the OE of MKS3 mutant rats versus controls (one picture shown per animal; n = 5 control, n = 4 mutant). (TIF) [file pone.0019694.s004.tif]

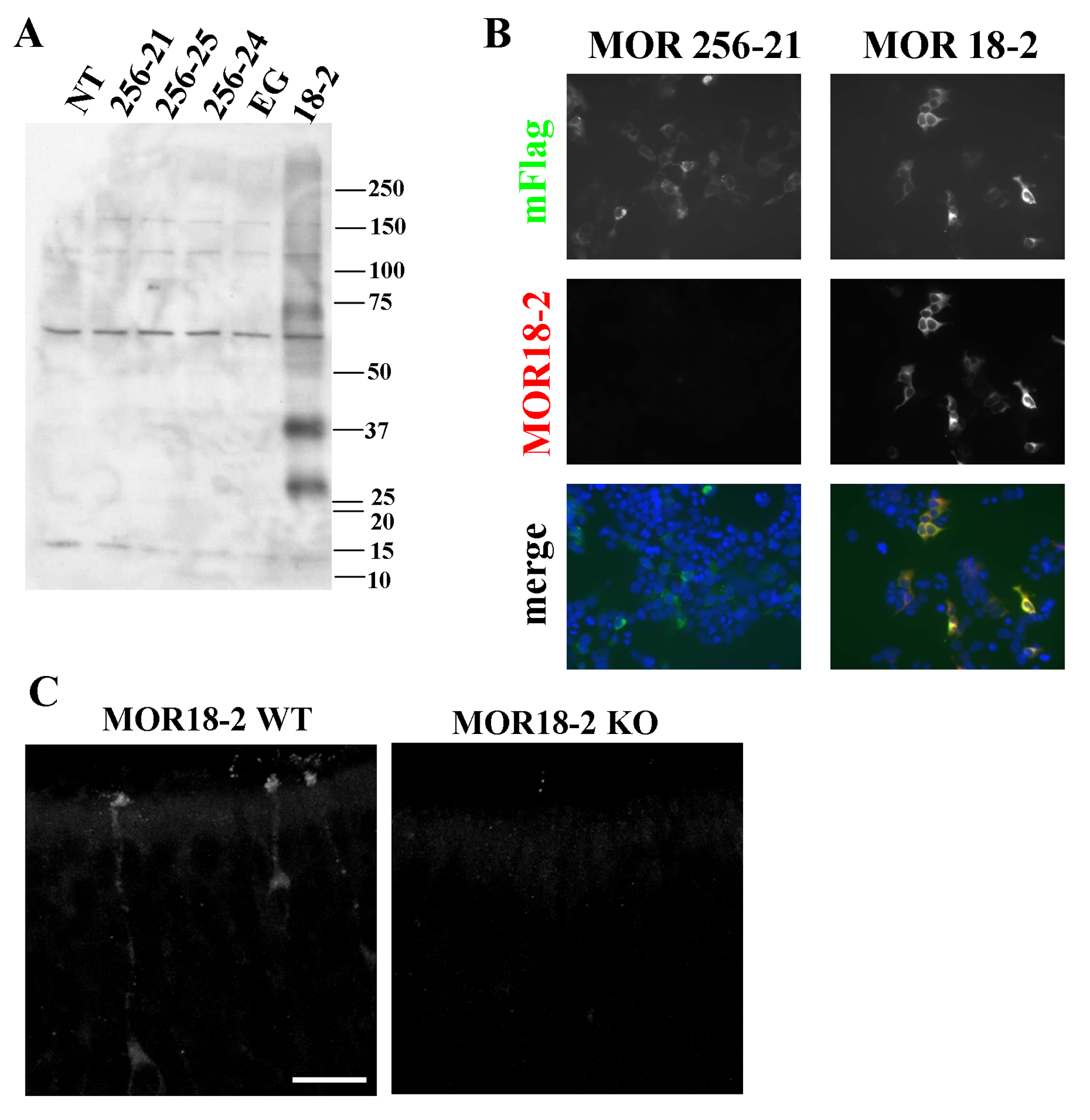

Supplement: Figure S5 — MOR18-2 antibody recognizes MOR18-2 protein in vitro and in vivo. A. Western blot of HEK 293T cells overexpressing various OR constructs. A band of the expected size (37 kDa), as well as other minor bands, were found only in cells overexpressing MOR18-2. B. Immunocytochemistry in HEK 293T cells using OR constructs containing an N-terminal Flag tag. Cells were transfected with MOR256-21 or MOR18-2 (as well as 256-25, 256-24, and EG – not shown). The MOR18-2 antibody specifically recognized MOR18-2, as shown by the colocalization of the MOR18-2 and monoFlag antibody signals. C. MOR18-2 recognizes zone 1 OSNs in MOR18-2+/+, but not MOR18-2−/− mice (Scale bar = 20 µm; compressed z-stacks). Although this antibody gives a specific signal in the OE, in other tissues tested it cross-reacts with an unknown protein (as evidenced by identical antibody staining patterns in wild-type and null mice). (TIF) [file pone.0019694.s005.tif]

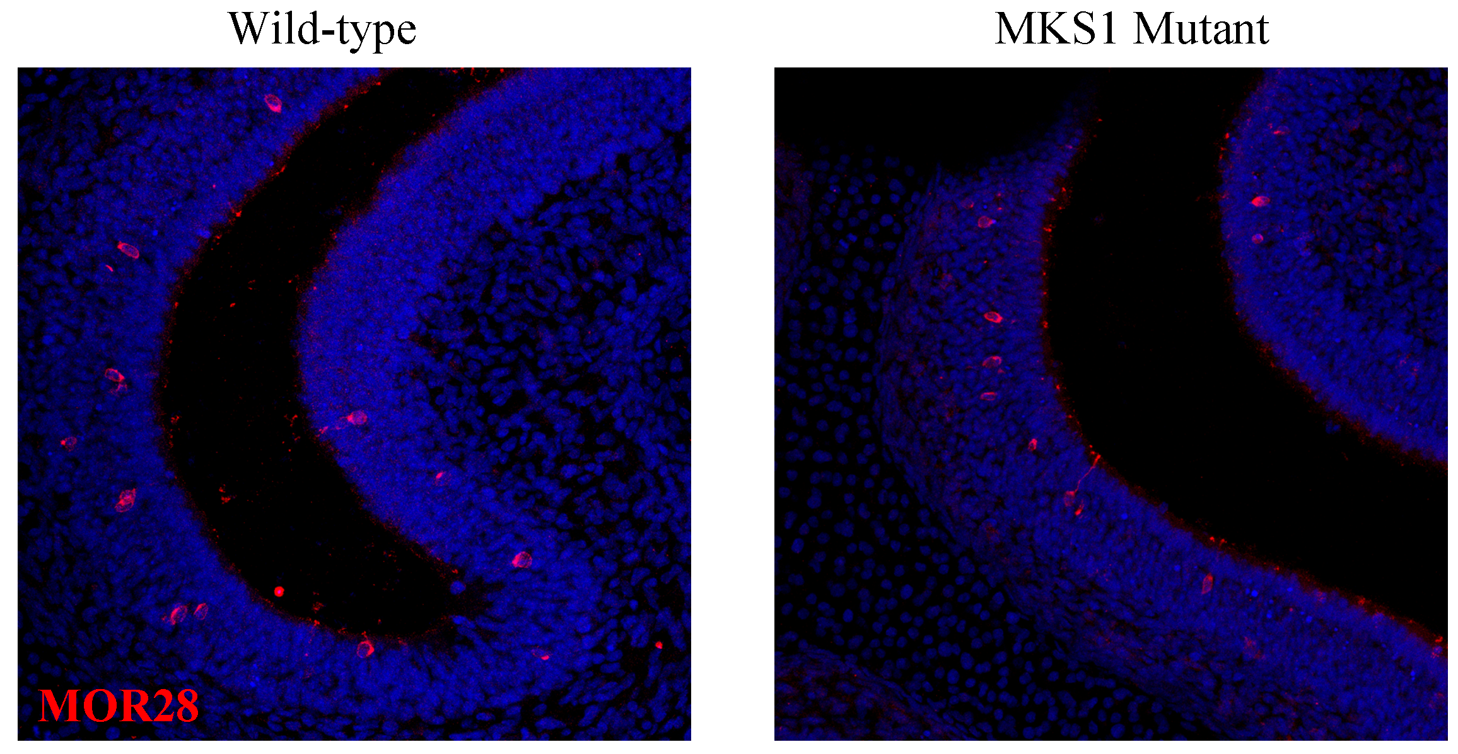

Supplement: Figure S6 — MOR28 zonal distribution is normal in MKS1 mutant mice. MOR28 staining is in red; nuclei shown in blue. (TIF) [file pone.0019694.s006.tif]

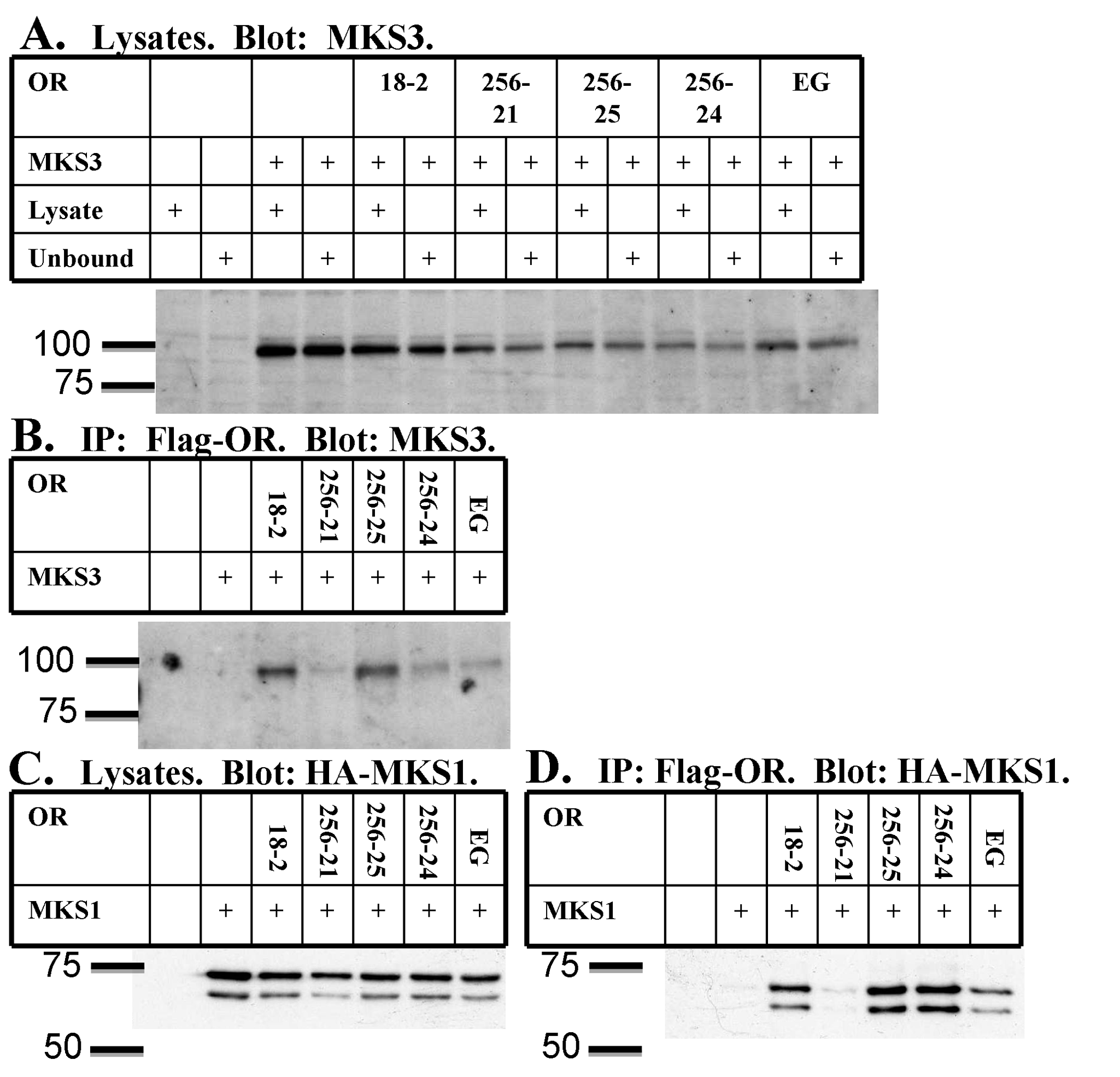

Supplement: Figure S7 — MKS3 and MKS1 both co-immunoprecipitate with OR constructs (molecular weight markers are indicated to the left of each blot). Flag-tagged OR constructs were co-transfected into COS cells along with MKS3, or HA-tagged MKS1. Lysates and unbound fractions are shown in Figure S6A (blotted with MKS3). For Figure S6B, immunoprecipitation was performed using a Flag antibody, and membranes were then blotted for MKS3. Co-expression of Flag-tagged MOR18-2, 256-21, 256-25, 256-24, and EG were all capable of facilitating the pull-down of MKS3, although the strongest signal was observed using MOR18-2 and 256-25. Figure S6C shows MKS1 lysates in the presence of various ORs, whereas Figure S6D shows the results of co-immunoprecipitation using a Flag antibody, followed by blotting for HA (MKS1). MKS1 also interacts with all of the ORs tested, with the strongest signal observed using MOR18-2, MOR256-25 and 256-24, and the weakest signal observed with MOR256-21. (TIF) [file pone.0019694.s007.tif]

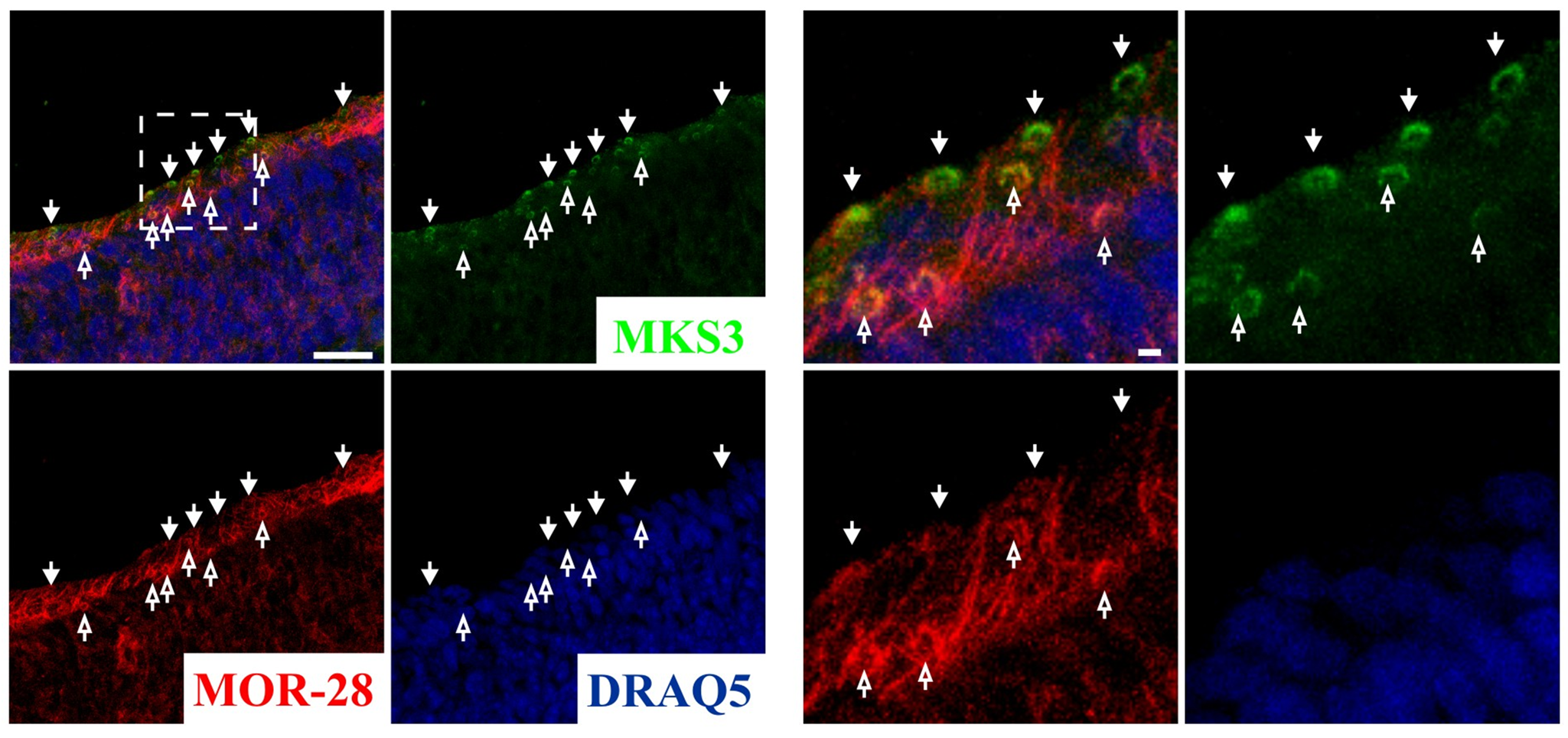

Supplement: Figure S8 — MOR28 and MKS3 colocalize in dendritic knobs. Knobs expressing MOR28 and MKS3 (open arrows), as well as knobs expressing MKS3 alone (filled arrows) were observed. This suggests that in OSNs, ORs and MKS3 are expressed in the same compartment. Red is MOR28; Green is MKS3; Blue is DRAQ5. The square in the left is shown at higher magnification in the right. Scale bar = 20 µm (left), 2 µm (right). (TIF) [file pone.0019694.s008.tif]

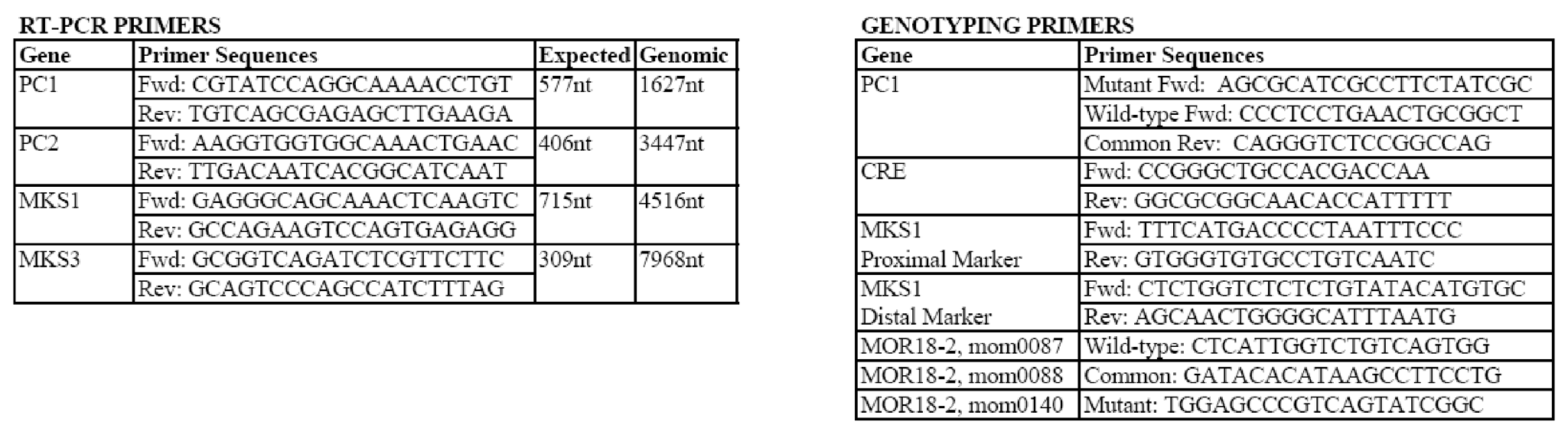

Supplement: Table S1 — Primer sequences for both RT-PCR and for genotyping are shown. (TIF) [file pone.0019694.s009.tif]
