# Supplementary material for: Renal Cystic Disease Proteins Play Critical Roles in the Organization of the Olfactory Epithelium
Source: PLoS One. 2011 May 13;6(5):e19694. doi: 10.1371/journal.pone.0019694 (PMC3094399; doi:10.1371/journal.pone.0019694)
Supplement: Text S1 — (PDF) [file pone.0019694.s010.pdf]

Supporting Information

Olfactory epithelium of $PC1^{-/-}$ mice

Because mutations in *PC1* are the most common cause of renal cystic disease, we also sought to determine if PC1 played a role in the OE. In order to examine this, we used a mouse model in which PC1 was specifically deleted in the mature OSNs using olfactory marker protein driving the expression of CRE (OMP-CRE).

In mice in which PC1 was deleted in the OE (*OMP-CRE, $PKD1^{flox/-}$*), we were unable to find differences in expression or localization of AC3, G_{olf} or MKS3 (Figure S3A). In addition, immunofluorescence for two well-characterized ORs (mOR28, M50) revealed apparently normal OR localization to the cilia, knob, apical dendrite and cell body; in addition, cilia structure appeared normal at the light microscopy level (Figure S3B).

Methods

Co-immunoprecipitation

COS cells were transfected with either empty vector (mock), or with vector encoding MKS3 (or, in other experiments, MKS1) +/- vector encoding MOR18-2, MOR256-21, MOR256-25, MOR256-24 or MOR-EG. The MKS3 construct (NM_153604.5) contains the full-length, untagged sequence of MKS3 in a pcDNA3.1 vector, and was modified from a vector originally made by C. Johnson. The MKS1 construct in pcDNA3.1 was provided by S. Weatherbee and includes a C-terminal HA

tag. All OR constructs were tagged with Flag and Rho. The MOR-EG construct (in a pME18S vector containing Flag and Rho tags) was a kind gift from S. Firestein; the other Olfr constructs were made by PCR of the full-length transcript from kidney using primers which contained restriction sites, and subcloning into the pME18S vector after excising the MOR-EG sequence with restriction enzymes. MOR18-2, MOR256-25 and MOR256-24 were subcloned between EcoRI and XhoI, whereas MOR256-21 was subcloned between EcoRI and SpeI. By immunofluorescence, we did not observe any changes in the localization of either protein (MKS or OR proteins) due to their co-expression in transfected cells.

Transfected cells were incubated for 24 hrs, and then lysed in cold TEN-T (100mM NaCl, 50mMTris pH=7.5, 1mM EDTA and 1% Triton X-100). Lysates were cleared by centrifugation, and 20μl were set aside as lysate controls. Lysates were then incubated overnight with 20μl washed mouse anti-Flag sepharose baeds. Beads were recovered by centrifugation, and 20μl of unbound lysate was set aside as representative of the unbound fraction. Beads were then washed (three times in TEN-T, once in PBS), and 3X sample buffer containing DTT was added. Samples were not heated. Samples were loaded onto 10% PAGE gels, and proteins were transferred to nitrocellulose. Membranes were probed using an anti-meckelin polyclonal antibody (MKS3) or an anti-HA antibody (MKS1) (1). The expected size for MKS3 is 112 kDa, whereas the expected size for MKS1 is 65 kDa. MKS1 typically ran as doublet.

References

1. Dawe HR et al (2007) The Meckel-Gruber Syndrome proteins MKS1 and meckelin interact and are required for primary cilium formation. *Hum Mol Genet* 2: 173-186.
